# Attitude to food supplement use: a survey promoted by the Italian Society of Pediatric Allergy and Immunology

**DOI:** 10.1186/s13052-024-01687-w

**Published:** 2024-06-20

**Authors:** Giorgio Ciprandi, Maria Daglia, Giulia Brindisi, Francesco Paolo Brunese, Giulio Dinardo, Alessandra Gori, Cristiana Indolfi, Matteo Naso, Enrico Tondina, Chiara Trincianti, Attilio Varricchio, Anna Maria Zicari, Michele Miraglia del Giudice, Lorenzo Drago

**Affiliations:** 1 Allergy Clinic, Casa di Cura Villa Montallegro, Genoa, Italy; 2 Department of Pharmacy, University of Napoli Federico II, Via D. Montesano 49, Naples, Italy; 3 International Research Center for Food Nutrition and Safety, Jiangsu University, Zhenjiang, China; 4 Department of Maternal Infantile and Urological Science, Sapienza University of Rome, Rome, Italy; 5 Primary Care Paediatrics, ASL Caserta, Caserta, Italy; 6 Department of Woman, Child and General and Specialized Surgery, University of Campania “Luigi Vanvitelli”, Naples, Italy; 7 Allergy Center, IRCCS Istituto Giannina Gaslini, Genoa, Italy; 8 Pediatric Clinic, Fondazione IRCCS Policlinico San Matteo, Pavia, Italy; 9 Department of Otolaryngology, University of Molise, Campobasso, Italy; 10 Laboratory of Clinical Microbiology & Microbiome, Department of Biomedical Sciences for Health, University of Milan, Milan, Italy; 11 UOC Laboratory of Clinical Medicine, IRCCS Multimedica Hospital, Milan, Italy

**Keywords:** Food supplements, Nutraceuticals, Survey, Pediatricians, Scientific society

## Abstract

Food supplements are defined as foodstuffs the purpose of which is to supplement the normal diet and which are concentrated sources of nutrients or other substances with a nutritional or physiological effect, often referred to as nutraceuticals, may exert benefit to the human body. Their use is increasing worldwide, including Europe and in Italy. However, some doctors are skeptical about their effectiveness and safety. This reluctance may depend on poor knowledge of the mechanisms of action and clinical evidence in literature. The Italian Society of Pediatric Allergy and Immunology (SIAIP) promoted the institution of an ad hoc Committee. The first initiative performed by this Committee was the administration of a questionnaire to the members of SIAIP.

The results of this survey provided interesting results. Most pediatricians know the food supplement concept but frequently need help understanding the mechanisms of action. Most prescribe food supplements, mainly for preventing infections or enhancing immune defense. In addition, they prefer to use food supplements as cycles or add-on therapy. Finally, most participants like to attend events on this issue and contribute to new evidence through trials.

In conclusion, this survey underscores the relevance of food supplement issues and attests to interest in this topic. However, there is a need to provide information and promote studies on this matter.

## Introduction

The term “nutraceutical” is a portmanteau of “nutrition” and “pharmaceutical.” It was coined to describe substances that possess health-promoting properties, and they are typically isolated from food sources and can be used as ingredients of enriched foods or food supplements. Therefore, nutraceuticals are products that combine the benefits of both nutrition and pharmaceuticals, as they are derived from food sources and provide health benefits beyond basic nutritional functions. These compounds have gained popularity for their potential to prevent, and manage various human diseases. Nutraceuticals can be classified into different categories based on their specific functions and applications in treating diseases. It’s important to note that while some nutraceuticals may have positive health effects, they are not a substitute for conventional medical treatments.

Food supplementsare defined as “foodstuffs the purpose of which is to supplement the normal diet and which are concentrated sources of nutrients or other substances with a nutritional or physiological effect, alone or in combination, marketed in dose form, namely forms such as capsules, pastilles, tablets, pills and other similar forms, sachets of powder, ampoules of liquids, drop dispensing bottles, and other similar forms of liquids and powders designed to be taken in measured small unit quantities” (DIRECTIVE 2002/46/EC OF THE EUROPEAN PARLIAMENT AND OF THE COUNCIL of 10 June 2002 on the approximation of the laws of the Member States relating to food supplement. OJEC. L 183/51).

The Table 1 summarizes the main nutraceuticals for human use and in some circumstances for treating human diseases. This list includes many substances, such as Antioxidants, compounds that help counteract the damaging effects of oxidative stress by neutralizing free radicals, Polyphenols, Plant-derived compounds with antioxidant properties and potential anti-inflammatory effects, Omega-3 Fatty Acids: Essential fatty acids with anti-inflammatory and cardiovascular benefits, Probiotics: Live microorganisms that confer health benefits when consumed in adequate amounts, Vitamin D: A fat-soluble vitamin important for bone health and immune system function, Glucosamine and Chondroitin: Compounds found in cartilage and commonly used for joint health [1–6]. .

**Table 1:** Synthetic list of the most common food supplements used in clinical practice

Antioxidants	Vitamin C, Vitamin E, Selenium, Zinc, Resveratrol, β-caroten
Polyphenols	Resveratrol, Quercetin, Catechins (green tea), Curcumin, Rosmarinic acid, Gingerol
ω−3 fatty acids	Fish oil, flaxseed oil
Probiotics	Lactobacilli, Bifidobacteria
Prebiotics	Inulin, FOS (fructo-olygosaccharides)
Symbiotics, Postbiotics	
Vitamin D3	
Glucosamine	
Chondroitin	
Immuno-modulants	Lactoferrin, Melatonin, Vitamin D3, Glucans
Anti-inflammatory	Resveratrol, Vitamin D3, Zinc, Glycyrrhetic acid, Lactoferrin, SCFA (short-chain fatty acids)

Nutraceuticals should complement, not replace, conventional medical approaches. Additionally, the field of nutraceutical research is dynamic, and new findings may influence recommendations over time.

Infectious diseases, especially those affecting the respiratory system, and allergic diseases, i.e., rhinitis, asthma, conjunctivitis, and food allergy, are among the most common illnesses during childhood. The management of these diseases is based on the use of drugs recommended in numerous guidelines, despite these drugs, due to the antimicrobial resistances burden, are not always completely effective or must be taken for prolonged periods. Therefore, it is not uncommon to observe adverse drug events or an increase of issues related to the antimicrobial resistant genes.

Moreover, a fair number of children tend to have frequent respiratory infections [7]. Finally, allergy sufferers are predisposed to frequent infections [8].

Allergic diseases and susceptibility to infections are conditions characterized by a functional defect in the immune system [9]. This defect can be remedied with evolution, but not always, and often, a remedy that can break the vicious circle between infections and allergies is required by the parents themselves.

For these reasons, it is common to resort to non-pharmacological remedies that can help resolve or at least accelerate the recovery from infections and reduce exacerbations of allergic symptoms. In this regard, many products of a non-pharmacological nature have long been available. These then include food supplements. Some compounds (i.e., zinc, Vitamin C and D, polyphenols, etc.) have demonstrated potential supportive roles in managing infections or boosting the immune system. Others (i.e., quercetin, Omega-3 Fatty Acids, but also Probiotics, Vitamin D, etc.) may play a role in supporting respiratory health and alleviating symptoms associated with respiratory allergic diseases.

The appropriateness of these food supplements may depend on individual health conditions, potential interactions with medications, and the specific nature of the allergic disease. Food supplements should complement, not replace, standard medical treatments prescribed by healthcare providers.

However, their consumption is constantly and progressively increasing because parents often self-prescribe them. On the contrary, several doctors are reluctant and skeptical about the actual efficacy of these products.

For this reason, the Italian Society of Pediatric Allergology and Immunology (SIAIP) has promoted an ad hoc commission to enhance scientific knowledge in this field.

The first act of this commission was to conduct a Survey administered to members of the scientific society.

## Methods

This Survey consists of a series of questions aimed at exploring the degree of knowledge of the subject and the attitude towards prescribing these products.

The organizational secretary of the SIAIP provided the list of participants. SIAIP members then received an e-mail invitation to participate in this Survey and, if interested, to link to an electronic platform expressly set up to collect responses.

The list of the various questions is shown in Table 2.

**Table 2:** Questionnaire with questions and answers, used in the present survey

Questions	Answer	Results
Your gender?	Female Male	64% 36%
What kind of structure do you work in?	University Hospital Primary care Private	32% 31% 25% 12%
What geographical area do you work in?	North-West Center North-East South Islands	30% 28% 18% 17% 7%
Do you know what nutraceuticals are?	Yes No	92% 8%
Do you know how nutraceuticals are regulated?	Yes No I don’t know	28% 50% 22%
Do you know of any potential risks associated with the use of nutraceuticals?	Yes No I don’t know	42% 40% 18%
Do you prescribe them in your clinical practice?	Yes No	59% 41%
If yes, in which therapeutic area?	Respiratory allergy Food allergy Any allergy Prevention of infections Drug enhancement Immunological defense	16% 3.5% 3.5% 36% 11% 30%
When using them, do you know the mechanisms related to nutraceuticals in depth?	Yes No	36% 64%
Do you think Nutraceuticals are really effective?	Yes No	72% 28%
Do you think that Nutraceuticals are safe?	Yes No	81% 19%
How do you use Nutraceuticals: Continuously?	Yes No	10% 90%
In cycles?	Yes No	67% 33%
Do you use Nutraceuticals as add-on therapy?	Yes No	55% 45%
Or as a single treatment?	Yes No	14% 86%
Do you think Nutraceuticals have a preventive effect on the development of allergies?	Yes No	28% 72%
Do you think Nutraceuticals have a preventive action against infections?	Yes No	63% 37%
Do you always use nutraceuticals in combination with conventional drug therapy?	Yes No	53% 47%
Would you like more information and insights into Nutraceuticals?	Yes No	91% 9%
Would you participate in Conferences, Courses, FAD on Nutraceuticals?	Yes No	80% 20%
Would you participate in studies (retrospective, interventional) on the use of Nutraceuticals?	Yes No	67% 33%

Globally, 127 members of the SIAIP participated in the Survey. The answers and results are reported in Table 2.

## Results

About 2/3 of the participants were female; most of them work in university or hospital structures (32% and 31%, respectively), 25% in primary care, and 12 in private clinics. A large number of the participants live in North-West and Central Italy.

The vast majority (92%) believe they know the meaning of the term food supplement and nutraceutical; however, most do not know the regulations on marketing these products.

Most participants (58%) do not know the potential risks of using food supplements.

Concerning the prescription, 59% of participants use food supplements in clinical practice. The most common indications include prevention of infections and immunological defense, followed by enhancement of pharmacological therapy and respiratory allergy.

Surprisingly, about two-thirds (64%) of participants admit not knowing the mechanisms of action of various nutraceuticals and food supplements in detail. Concerning the perception of the actual effectiveness of food supplements, 72% of colleagues believe that food supplements are really effective; moreover, 81% believe that food supplements are also safe.

As regards the modes of use, only 10% of participants prescribe food supplements continuously, and 67% in cycles. In addition, 55% use food supplements as add-on therapy, whereas only 14% as single treatment.

A few participants (28%) think that food supplements may really prevent the development of allergic disorders; contrarily, 63% hold to be true that food supplements prevent infections.

Interestingly, 91% of participants would like more information and insights into nutraceuticals and food supplements, 80% participate in events on this topic, and 67 participate in studies.

These findings are fascinating and noteworthy as they reflect what many doctors and members of the SIAIP consider the nutraceutical world.

The most relevant outcomes confirm that almost all (92%) declare knowing nutraceuticals and food supplements, even if, successively, most of them do not know the regulation and, overall, the exact mechanism of action. Accordingly, most participants would like to achieve more information and insights about nutraceuticals and food supplements through educational programs and participation in trials.

From a clinical point of view, enhancing immunological response and preventing infections represent the most common indications. In addition, the cyclic prescription and add-on therapy are the most frequent modes of use.

## Discussion

This Survey presents some limitations, including the relatively limited number of participants and the need for more validation of this Survey. However, the results reflect what happens in clinical practice and mirror the real attitude in prescribing food supplements.

It is well known that the and food supplement issue is debated and controversial as some doctors consider inappropriate the use of food supplements in clinical practice. The main reason for this aversion against food supplements depends on the awareness that there is a lack of evidence about their efficacy and safety. In this regard, a recent consensus provided a negative consideration of nutraceuticals in preventing recurrent respiratory infections in children [10]. However, despite much evidence about its preventive and adjunctive efficacy, vitamin D did not achieve recommendations.

However, the methodology used could be inadequate to evaluate these products as most studies were conducted in real life. Namely, it is challenging and costly to conduct randomized controlled clinical trials. Instead, more appropriate and valuable are studies conducted in real life, which make it possible to recruit many patients who, among other things, are not mainly selected and thus offer an accurate mirror of what happens in everyday clinical practice.

Moreover, the scarce attitude to use food supplements may also derive from the poor knowledge of these products. Undoubtedly, the number of products is vast. However, it is essential to consider a few considerations before using them. In particular, doctors should know the exact composition of such a product, the quantity of each component, the standardization and purification of ingredients, and the safety. Recently, some studies summarized the evidence concerning the use of non-pharmacological remedies in managing children with allergy, asthma, and infections [11–14]. As a result, there is convincing evidence that selected products have adequate demonstration of effectiveness and safety. Moreover, it is relevant to underline the safety issue as food supplements can be associated with adverse reactions if used behind their indications, at high doses, mixed with incompatible components. In this regard, when prescribing food supplements, the doctor should be aware of knowing the indication, the composition (data on source, extraction, purification, production, stability), the evidence of efficacy and safety of the specific product, and the duration of treatment.

In conclusion, this survey underlines the need to create culture around this topic. In this regard, the SIAIP is honored to fulfill this information mission and has set up a special committee responsible for disseminating information and preparing articles and studies on this topic.

## Data Availability

All data generated and analysed during this survey are included in this published article.

## Abbreviations

SIAIP: Società italiana di allergologia ed immunologia pediatrica

## References

[1] Shaubhik Anand and Navneeta Bharadvaja (2022). Potential benefits of nutraceuticals for oxidative stress management. Rev Bras Farmacogn.

[2] Pandey KB, Rizvi SI (2009). Plant polyphenols as dietary antioxidants in human health and disease. Oxid Med Cell Longev.

[3] Mozaffarian D, Wu JH (2011). Omega-3 fatty acids and cardiovascular disease: effects on risk factors, molecular pathways, and clinical events. J Am Coll Cardiol.

[4] Hill et al. 2014. Expert Consensus Document: The International Scientific Association for Probiotics and Prebiotics Consensus Statement on the Scope and Appropriate Use of the Term Probiotic. Nat Rev Gastroenterol Hepatol. 2014;11(8):506 – 14. 10.1038/nrgastro.2014.66 24912386

[5] Holick. 2007. Vitamin D Deficiency. N Engl J Med. 2007;357(3):266 – 81. 10.1056/NEJMra070553 17634462

[6] De Los Reyes GC, Koda RT, Lien EJ (2000). Glucosamine and chondroitin sulfates in the treatment of osteoarthritis: a survey. Prog Drug Res.

[7] de Benedictis FM, Bush A (2018). Recurrent lower respiratory tract infections in children. BMJ.

[8] Klimek L (2022). Immune system and allergies-An unholy alliance]. Internist (Berl).

[9] Ogulur I, Pat Y, Ardicli O, Barletta E, Cevhertas L, Fernandez-Santamaria R (2021). Advances and highlights in biomarkers of allergic diseases. Allergy.

[10] Chiappini E, Santamaria F, Marseglia GL, Marchisio P, Galli L, Cutrera R (2021). Prevention of recurrent respiratory infections: inter-society Consensus. Ital J Pediatr.

[11] Murgia V, Ciprandi G, Votto M, De Filippo M, Tosca MA, Marseglia GL (2021). Natural remedies for acute post-viral cough in children. Allergol Immunopathol (Madr).

[12] Ciprandi G (2023). Self-management in allergic rhinitis: strategies, outcomes and integration into Clinical Care. J Asthma Allergy.

[13] Trincianti C, Tosca MA, Ciprandi G (2023). Updates in the diagnosis and practical management of allergic rhinitis. Expert Rev Clin Pharmacol.

[14] Ciprandi G, Tosca MA (2022). Probiotics in Children with Asthma. Child (Basel).

